# Universal approach for full endoscopic decompression and percutaneous transpedicular fixation of the lumbar spine: A medial central approach (ACM)

**DOI:** 10.1097/MD.0000000000026310

**Published:** 2021-06-11

**Authors:** Víctor Hugo Malo-Camacho, Gerardo Enrique Bañuelos-Díaz, Víctor Hugo Martínez-Velázquez, Luis López-Ortega, Oscar Malo-Macías, Enrique Villarreal-Ríos, Alejandro Sosa-Gallegos, Mauricio Alva-Nájera, Mario Iván Mejía-Valencia

**Affiliations:** a Instituto de Columna Newro Spine; b Hospital General de Querétaro, Secretaría de Salud del Estado de Querétaro, Querétaro, Mexico.

**Keywords:** endoscopy, fixation, lumbar

## Abstract

Cohort study.

This study aimed to determine the effectiveness of the universal approach of full endoscopy and percutaneous transpedicular fixation via a medial central approach (ACM) performed to surgically treat patients with lumbar degenerative surgical pathologies.

Alternatives to interventionist treatments available to patients with lumbar degenerative surgical pathologies are related to recovery from minimally invasive surgery. Considering this, full endoscopic spinal decompression (full endoscopy) and percutaneous transpedicular fixation via an ACM represent advances in neurosurgical procedures, in particular, spinal surgery. Thus, the introduction of endoscopic and minimally invasive surgeries for the lumbar region has become 1 of the most important advances in modern surgery.

A cohort of 79 patients undergoing full endoscopy and percutaneous transpedicular fixation was evaluated 6 times in 1 year. Pain intensity was measured using the visual analog scale (VAS), and lumbar functionality was measured using the Oswestry Disability Index (ODI). Six evaluations were performed: before surgery and on discharge after surgery as well as at 1, 3, 6, and 12 months after surgery.

Before the ACM was applied, the VAS pain score was 8.52. At 11 hours post-surgery, the pain score reduced to 2.59 points (a difference of 5.73 points; *P* = 0.001). Of the 10 ODI domains evaluated, a difference was found between the period prior to surgery and 1 month later (*P* < 0.01).

The universal approach to full endoscopy and lumbar percutaneous transpedicular fixation via an ACM is highly effective for patients with lumbar surgical degenerative pathologies.

## 1 Introduction

Lower back pain is a significant global health problem that affects individuals and greatly reduces productivity. Clinicians have therefore been strongly motivated to seek out novel medical and surgical management strategies for the same.^[1–5]^ The use of invasive surgery as an intervention harms the integrity of the patient and often has adverse consequences.^[6]^ The use of traditional open surgical techniques often results in a slow recovery process, including slow remission of the symptoms that surgery seeks to mitigate.^[7,8]^ Given this scenario, the emergence of endoscopic and minimally invasive surgeries for the lower back (including neurosurgical procedures, particularly those for the spine) has become 1 of the most important advances in modern surgery.^[9]^

Minimally invasive endoscopic surgery in the lumbar spine minimizes surgical costs and time; it also lowers the rate of complications such as infection and bleeding. In the clinical setup, minimally invasive surgery decreases pain and increases the chances of patient mobility, including performing daily activities.^[9,10]^

The visual analog scale (VAS)^[11]^ is used to measure pain, and the Oswestry Lumbar Pain Disability Questionnaire, also known as the Oswestry Disability Index (ODI), is used to evaluate additional clinical aspects.^[12,13]^

In this context, this study aimed to determine the effectiveness of the universal approach for full endoscopic decompression (full endoscopy) and percutaneous transpedicular fixation of the lumbar spine via a medial central approach (ACM) among patients with lumbar degenerative surgical pathologies who require spinal decompression and fixation.

## 2 Methods

### 2.1 Design

This cohort included patients with a lumbar degenerative surgical pathology who requested medical care for their pain. The control group are the same patients before surgery, that the after surgery. The same neurosurgeon who prescribed decompression and fixation evaluated all patients. A universal approach was used for full endoscopy and percutaneous transpedicular fixation of the lumbar spine via an ACM was performed for this sample. Prior to inclusion in the study, clinical evaluation of each patient was performed before surgery, on discharge, and at 1, 3, 6, and 12 months after surgery, in all patients, the assessment of pain and disability was performed by the same doctor. The patients were treated at a medical service center in Querétaro, México. The follow-up of each patient occurred over 12 months, but cohort enrolment spanned 3 years, from January 2016 to December 2018.

### 2.2 Patients’ indications for fixation

Patients without a history of spinal surgery who continued pain management after surgery for 1 year were included in the study. Patients with axial and radicular pain and segmental instability as determined through radiology were also included. Patients with osteoporosis, osseous metabolic alterations, and psychological conditions and those who lack clinico-radiological correlation data were excluded from the study.

The criteria for performing transpedicular fusion are radiological data of instability, radicular pain associated with instability, radicular pain, and pain in the lower back associated with instability. The diagnostic criteria for spondylolisthesis are herniated disc with instability, narrow lumbar canal with instability, and unstable degenerative scoliosis with narrow lumbar canal, and data corroborated with radiology and nuclear magnetic resonance studies.

These patients underwent full endoscopy and percutaneous transpedicular fixation of the lumbar spine via an ACM that was composed of 7 stages.

### 2.3 Technique: full endoscopy and percutaneous transpedicular fixation of the lumbar spine via an ACM

We used the Endospine endoscope (Destandau, Storz). Usually, lateral ports are used for the placement of percutaneous transpedicular screws; here, we used midline ports for placement. These same ports were used to perform the full endoscopy for various decompressions. The surgical site was identified using fluoroscopy under intravenous general anesthesia with the patient in the ventral decubitus position.

**Figure 1:**
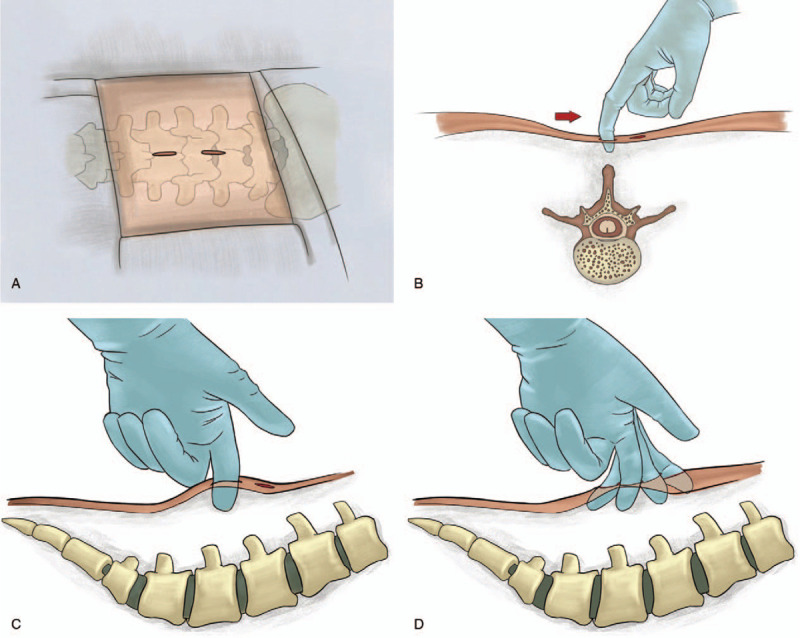
Skin incision and detachment.

**Stage 1.** The incision was made 1.0 to 1.5 cm on the midline. We detached the skin 2.0 to 3.0 cm around the incision, enabling us to manipulate this approach in the desired direction according to the pathology (Fig. 1).

**Stage 2.** Subsequently, the fascia was opened through monopole cautery, and an incision was made through the paravertebral muscle in the direction of the muscle fibers until the lamina of the surgical site that was to be decompressed was reached.

**Figure 2:**
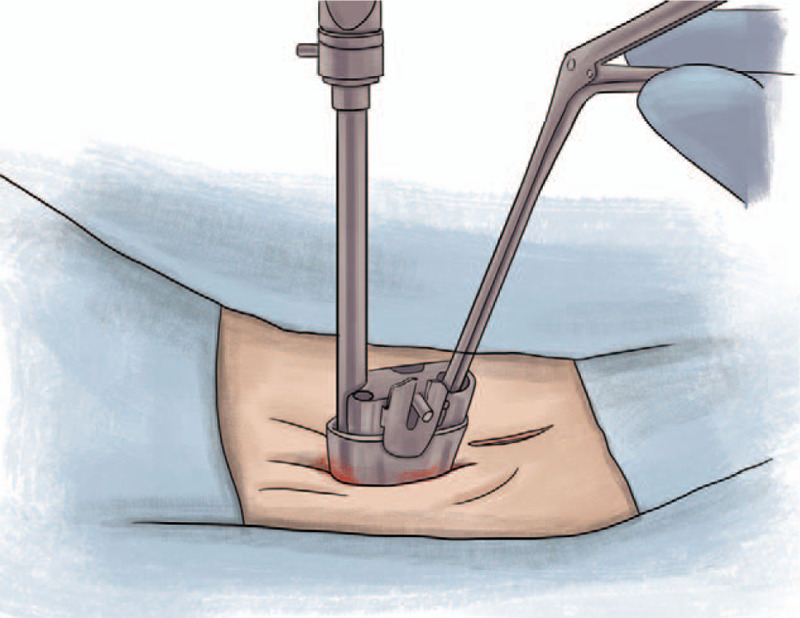
Cleaning of the surgical site in the region of the lamina and placement of endoscopic trocar.

**Stage 3**. The surgical site was cleaned in the region of the lamina, and the endoscopic trocar was placed to start surgery using an endoscopic approach (Fig. 2).

**Stage 4.** A partial laminectomy was started at the surgical site; a drill was occasionally used to enable quicker sub-laminar access. Subsequently, routine surgery commenced with endoscopic visualization depending on the procedure to be performed, such as in herniated disk, lumbar spinal stenosis at 1 or multiple levels, or unilateral or bilateral decompression.

**Figure 3:**
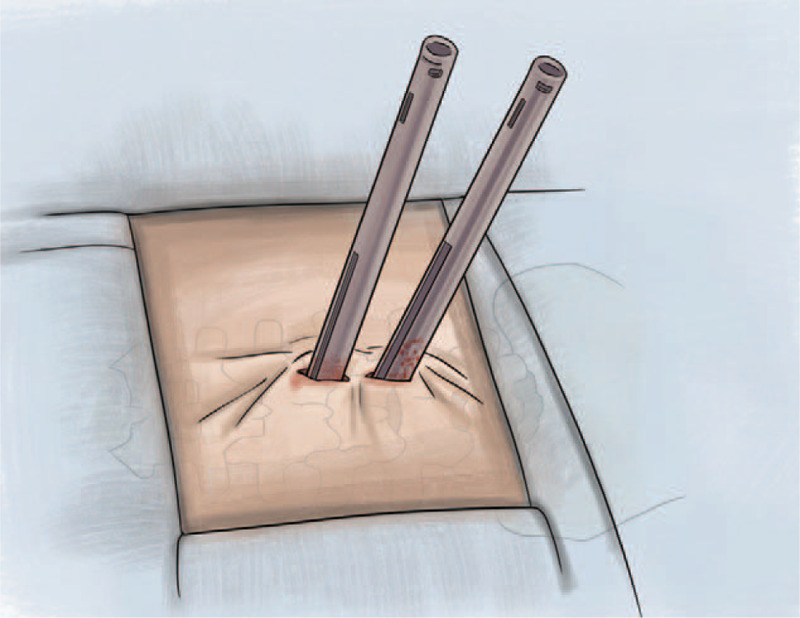
The same medial approach.

**Stage 5**. Bilateral lumbar spine decompression can be performed on the same side where the initial approach is performed, or it can be performed from the contralateral side. Regardless of which side, the same medial approach port is used. Moreover, decompressions can be performed up to 2 levels with a single port (Fig. 3).

**Figure 4:**
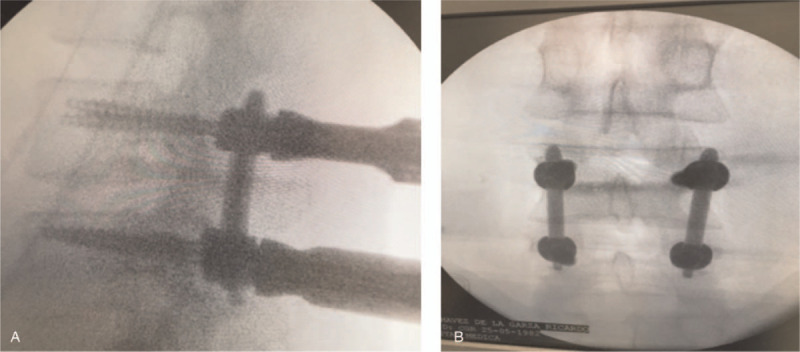
Placement of screws.

**Stage 6.** The same midline approach was used to place the percutaneous screws measuring between 1.0 and 1.5 cm, placing 1 side at a time. For this purpose, only the cutaneous approach is moved to the screw placement site. Importantly, screw placement was initially performed ipsilaterally until the bar was placed. Then, the screws were placed contralaterally using the same midline approach port of 1.0 and 1.5 cm. A posterolateral bone matrix was placed at the level of the facet joints (Fig. 4).

**Stage 7**. The fascia and cellular tissue were closed with vicryl, and the skin was closed with a subdermal monocryl. The entire procedure was performed under continuous intraoperative neuromonitoring, and each screw was verified after placement.

### 2.4 Sample size

The sample size was using the formula of average for 2 populations, with a confidence level of 95% (Zalfa = 1.96), power of the test of 80% (Zbeta = 0.84), assuming that the pain level (measured on a VAS scale) before surgery was 8 (μ_0_ = 8.00) standard deviation 1.60 (σ_0_ = 1.60) and 24 hours after surgery was 7 (μ_1_ = 7.00), and standard deviation 1.6 (σ_1_ = 1.60). The calculated size corresponded to 35.44, however, we worked with a sample size of 79 (n = 79). The sampling technique was by consecutive cases, using the list of patients who sought medical attention as the sampling frame.

#### 2.4.1 Variables

The study variables were age, sex, preoperative diagnosis, type of anesthesia, amount of transoperative bleeding, and length of hospital stay.

### 2.5 Measurements

#### 2.5.1 Pain

To determine the effectiveness of the full endoscopy and percutaneous transpedicular fixation via an ACM, the VAS^[11]^ was used. This scale was used to identify the intensity of pain from 0 to 10 from the patient's perspective, where 0 denotes the absence of pain and 10 corresponds to maximal pain. In this case, the evaluation was performed before surgery, immediately after surgery, and at 1, 3, 6, and 12 months after surgery.

### 2.6 Activities of daily living

The effectiveness of full endoscopy and percutaneous transpedicular fixation via an ACM was also evaluated using the ODI.^[12,13]^ This instrument evaluates 10 domains from the patient's perspective, including pain intensity as well as the ability to perform personal care, lift, walk, sit, stand, sleep, have sex, maintain one's social circle, and travel. The evaluation was performed using ordinal scales ranging from 0 to 5, where 0 denotes the best possible condition and 5 represents the worst possible condition. The evaluation was performed 5 times: before surgery and at 1, 3, 6, and 12 months after surgery.

The physiotherapist who evaluated the patients by applying the VAS^[11]^ and ODI^[12,13]^ had undergone training in the management of these instruments.

### 2.7 Statistical analyses

The variables of sex, type of diagnosis, and type of anesthesia were analyzed with percentages and confidence intervals for percentages; age, amount of bleeding during surgery, and days of hospitalization were analyzed with averages and confidence intervals for averages; the pain variable, measured on the VAS scale, was evaluated at 6 different times, before surgery, at 24 hours, at 1 month, at 3 months, 6 months, and 12 months; the Kolmogorov–Smirnov test was applied, the result showed no normality; consequently, the Friedman test was used to compare the 6 evaluations as a whole and the Wilcoxon test was used to compare in pairs; disability was evaluated with the ODI scale in 10 dimensions, in this variable the Kolmogorov–Smirnov test did not show normal distribution, consequently, the Friedman test was used to compare the 6 evaluations.

#### 2.7.1 Ethics

The manuscript was not originally raised as a research project. The manuscript is a product of medical practice; however, the project adhered to the recommendations of the Institutional Review Board, the surgical procedure is approved as a treatment and the benefits obtained are superior, the intervention did not unnecessarily expose the patient to risk, the informed consent of the patient was obtained To perform the surgical technique, the anonymity of the patients was maintained and the information was protected and encrypted by the project leader.

#### 2.7.2 Funds

This work was based on daily clinical practice, and no specific funding was provided.

## 3 Results

The mean age of the study population was 52.02 years (95% CI 48.59–55.45), and men comprised 51.9% of the sample (95% CI 40.6. 63.1).

The most frequent preoperative diagnosis was lumbar spinal stenosis (79.7%; 95% CI 70.8–88.6). Table 1 shows the remaining information.

**Table 1:** Preoperative diagnosis of the sample.

		95% CIs	
Preoperative diagnosis	Percentage	Lower	Upper
Lumbar spinal stenosis	79.7	70.8	88.6
Spondylolisthesis	20.3	11.7	29.5

CI = confidence interval.

General anesthesia was used in 91.1% (95% CI 84.8–97.4) of the patients, 50.37 mL (95% CI 49.94–50.81) of intraoperative bleeding was reported, and the mean hospital stay was 11.62 hours (95% CI 11.15–12.09). In all cases, the patient was managed with ketorolac when discharged from the hospital.

Before endoscopy via an ACM, the VAS pain score was 8.52 points; at hospital discharge, it was 11.62; and hours later, it was 2.59 points. This difference of 5.73 points was significant (*P* < 0.001). A significant change of 1.73 points was also found between the post-surgery stage and 1 month after surgery (*P* < 0.001). Although the change in pain intensity 1 month after surgery was not significant, pain levels at 1 month post-surgery were close to 0, an ideal value (Table 2).

**Table 2:** Evaluation of pain and change in pain before and after surgery as well as 1, 3, 6, and 12 mo later using the VAS.

Pain comparison				
Stage	Mean	Standard deviation	Friedman	*P*
Before	8.32	1.97	320.55	0.001
After	2.59	2.11		
1 mo	0.86	1.40		
3 mo	0.82	1.23		
6 mo	0.84	1.10		
12 mo	0.82	1.11		

Change in pain comparison									
Before vs after		After vs 1 mo		1 mo vs 3 mo		3 mo vs 6 mo		6 mo vs 12 mo	
Difference in score	*P*	Difference in score	*P*	Difference in score	*P*	Difference in score	*P*	Difference in score	*P*
5.73	0.001	1.73	0.001	0.04	0.257	0.02	0.796	0.02	0.564

The VAS ranges from 0 to 10, where 0 corresponds to the absence of pain and 10 denotes the maximum pain level. The change in pain was compared using the Wilcoxon test. VAS = visual analog scale.

At 12 months after endoscopy via an ACM, the VAS pain score was 0.82 points, representing a total decrease of 7.50 points.

All 10 ODI domains significantly improved (*P* < 0.001). Pain was associated with the highest score before surgery (3.41); at 12 months later, the score decreased to 0.03, and this difference was significant (*P* < 0.001). Table 3 shows the score of each domain for the 5 periods evaluated.

**Table 3:** Evaluation of clinical characteristics before surgery and 1, 3, 6, and 12 mo after surgery using the Oswestry Disability Index.

	Time of evaluation						
Domain	Before surgery	1 mo after surgery	3 mo after surgery	6 mo after surgery	12 mo after surgery	Friedman	*P*
Pain intensity	3.41	1.04	1.00	0.03	0.03	295.12	0.001
Personal care	2.48	1.08	1.01	0.03	0.03	257.18	0.001
Lifting	3.15	1.43	1.01	0.01	0.01	295.98	0.001
Walking	2.78	1.16	1.15	0.01	0.01	282.69	0.001
Sitting	3.20	1.18	1.06	0.04	0.04	289.72	0.001
Standing	3.03	1.22	1.01	0.01	0.01	291.37	0.001
Sleeping	2.62	1.15	1.00	0.03	0.03	273.27	0.001
Sex life	1.01	0.37	0.34	0.00	0.00	108.69	0.001
Social life	3.08	1.05	1.01	0.01	0.01	276.65	0.001
Travelling	2.99	1.11	0.99	0.01	0.01	280.13	0.001

The Oswestry Disability Index is measured from 0 to 5, where 0 denotes the best condition and 5 represents the worst possible condition.

Significant differences were found between the periods prior to surgery and immediately after surgery for all domains (*P* < 0.001). When the evaluation 1 month after surgery was compared with that at 3 months later, no differences were found, except for an improvement in lifting (*P* < 0.001; Table 4).

**Table 4:** Comparisons of clinical characteristics between study periods.

	Before vs 1 mo		1 mo vs 3 mo		3 mo vs 6 mo		6 mo vs 12 mo	
Domain	Difference in score	*P*	Difference in score	*P*	Difference in score	*P*	Difference in score	*P*
Pain intensity	2.37	0.001	0.04	0.090	0.97	0.001	0.00	1.00
Personal care	1.40	0.001	0.07	0.564	0.98	0.001	0.00	1.00
Lifting	1.72	0.001	0.42	0.001	1.00	0.001	0.00	1.00
Walking	1.62	0.001	0.01	0.799	1.14	0.001	0.00	1.00
Sitting	2.02	0.001	0.12	0.188	1.02	0.001	0.00	1.00
Standing	1.81	0.001	0.21	0.062	1.00	0.001	0.00	1.00
Sleeping	1.47	0.001	0.15	0.041	0.97	0.001	0.00	1.00
Sex life	0.64	0.001	0.03	0.317	0.34	0.001	0.00	1.00
Social life	2.03	0.001	0.04	0.581	1.00	0.001	0.00	1.00
Travelling	1.88	0.001	0.12	0.196	0.98	0.001	0.00	1.00

An improvement was also found when the 3- and 6-month evaluations were compared (*P* < 0.001; Table 4). No significant change was found in any of the domains between 6 and 12 months.

Pre-surgery restrictive pain was the most common for all domains, but this trend was reversed 1 month after surgery when most scores corresponded to ideal pain levels. Table 5 shows the results of the categories across the 6 evaluations performed for each domain.

**Table 5:** Prevalence of each domain item by the moment of evaluation.

	Moment of evaluation Prevalence				
Item and domain	Before	1 month	3 months	6 months	12 months
Pain intensity					
I can endure pain without needing painkillers	3.8	1.3	100.0	97.5	97.5
The pain is strong, but I can manage it without taking painkillers	2.5	94.9	0.0	2.5	2.5
Painkillers completely relieve the pain	6.3	2.5	0.0	0.0	0.0
Painkillers relieve some of the pain	39.2	1.3	0.0	0.0	0.0
Painkillers barely relieve the pain	32.9	0.0	0.0	0.0	0.0
Painkillers do not relieve the pain, and I don’t take them	15.2	0.0	0.0	0.0	0.0
Personal care					
I can look after myself normally without causing extra pain	12.7	1.3	1.3	97.5	97.5
I can look after myself normally, but it causes extra pain	13.9	92.4	96.2	2.5	2.5
It is painful to look after myself, and I am slow and careful	12.7	5.1	2.5	0.0	0.0
I need some help, but I can manage most of my personal care	38.0	1.3	0.0	0.0	0.0
I need help every day with most aspects of self-care	19.0	0.0	0.0	0.0	0.0
I do not get dressed, I wash with difficulty, and I stay in bed	3.8	0.0	0.0	0.0	0.0
Lifting					
I can lift heavy weights without extra pain	1.3	1.3	1.3	98.7	98.7
I can lift heavy weights, but it increases pain	8.9	74.7	96.2	1.3	1.3
Pain prevents me from lifting heavy weights off the floor, but I can manage if they are conveniently placed (e.g., on a table)	15.2	6.3	2.5	0.0	0.0
Pain prevents me from lifting heavy weights, but I can manage to lift medium weights if they are conveniently positioned	32.9	15.2	0.0	0.0	0.0
I can lift very light weights	31.6	2.5	0.0	0.0	0.0
I cannot lift or carry anything at all	10.1	0.0	0.0	0.0	0.0
Walking					
Pain does not prevent me from walking any distance	5.1	0.0	1.3	98.7	98.7
Pain prevents me from walking more than 1 kilometer	11.4	87.3	88.6	1.3	1.3
Pain prevents me from walking more than 500 meters	8.9	8.9	3.8	0.0	0.0
Pain prevents me from walking more than 250 meters	53.2	3.8	6.3	0.0	0.0
I can only walk with a cane or crutches	17.7	0.0	0.0	0.0	0.0
I am in bed most of the time, and I have to crawl to the bathroom	3.8	0.0	0.0	0.0	0.0
Sitting					
I can sit in any chair as long as I like	2.5	1.3	1.3	96.2	96.2
I can only sit in my favorite chair as long as I like	10.1	84.8	93.7	3.8	3.8
Pain prevents me from sitting more than 1 hour	15.2	10.1	2.5	0.0	0.0
Pain prevents me from sitting more than 30 minutes	19.0	2.5	2.5	0.0	0.0
Pain prevents me from sitting more than 10 minutes	43.0	1.3	0.0	0.0	0.0
Pain prevents me from sitting at all	10.1		0.0	0.0	0.0
Standing					
I can stand for as long as I want without extra pain	3.8	1.3	1.3	98.7	98.7
I can stand as long as I want, but it gives me extra pain	5.1	82.3	96.2	1.3	1.3
Pain prevents me from standing for more than 1 hour	15.2	11.4	2.5	0.0	0.0
Pain prevents me from standing for more than 30 minutes	43.0	3.8	0.0	0.0	0.0
Pain prevents me from standing for more than 10 minutes	26.6	1.3	0.0	0.0	0.0
Pain prevents me from standing at all	6.3	0.0	0.0	0.0	0.0
Sleeping					
My sleep is never disturbed by pain	7.6	1.3	1.3	97.5	97.5
My sleep is occasionally disturbed by pain	12.7	92.4	97.5	2.5	2.5
Because of pain, I obtain less than 6 hours of sleep	22.8	1.3	1.3	0.0	0.0
Because of pain, I obtain less than 4 hours of sleep	30.4	1.3	0.0	0.0	0.0
Because of pain, I obtain less than 2 hours of sleep	20.3	2.5	0.0	0.0	0.0
Pain prevents me from sleeping at all	6.3	1.3	0.0	0.0	0.0
Sex life					
My sex life is normal and causes no extra pain	59.5	63.3	65.8	100.0	100.0
My sex life is normal but causes some extra pain	5.1	36.7	34.2	0.0	0.0
My sex life is nearly normal but is very painful	13.9	0.0	0.0	0.0	0.0
My sex life is severely restricted by pain	17.7	0.0	0.0	0.0	0.0
My sex life is nearly absent because of pain	3.8	0.0	0.0	0.0	0.0
Pain prevents me from having any sex life at all	0.0	0.0	0.0	0.0	0.0
Social life					
My social life is normal and gives me no extra pain	8.9	1.3	1.3	98.7	98.7
My social life is normal but increases the degree of pain	3.8	94.9	97.5	1.3	1.3
Pain has not significantly affected my social life, apart from limiting my more energetic interests (e.g., dancing and so on)	3.8	1.3	1.3	0.0	0.0
Pain has restricted my social life, and I do not go out often	46.8	2.5	0.0	0.0	0.0
Pain has restricted my social life to my home	27.8	0.0	0.0	0.0	0.0
I have no social life because of pain	8.9	0.0	0.0	0.0	0.0
Travelling					
I can travel anywhere without pain	7.6	96.2	1.3	98.7	98.7
I can travel anywhere but it gives me extra pain	6.3	1.3	98.7	1.3	1.3
My pain is bad, but I manage journeys over 2 hours	12.7	1.3	0.0	0.0	0.0
Pain restricts me to journeys of less than 1 hour	35.4	1.3	0.0	0.0	0.0
Pain restricts me to short necessary journeys under 30 minutes	29.1	0.0	0.0	0.0	0.0
Pain prevents me from traveling except for going to the doctor or hospital	8.9	0.0	0.0	0.0	0.0

## 4 Discussion

The trend toward chronic degenerative diseases is increasing due to lifestyle and demographic transitions. Within the context of accelerated scientific and technological advances,^[14,15]^ alternatives for managing morbid processes must evolve at the same rate for quick incorporation into an individual's social dynamics to maintain the standards of living. Open surgery is universally used to achieve spinal decompression and fixation, and lateral ports are used to place percutaneous transpedicular screws. This article demonstrates the effectiveness of using only midline ports as a universal approach and the same medial ports for full endoscopy and percutaneous transpedicular fixation via an ACM for different decompressions and fixations.

Thus, this study is important because it shows the results of the above approach among patients with lumbar degenerative surgical pathologies who meet the criteria for surgical decompression and transpedicular fixation according to the existing literature.

The indication to perform ACM is patients who require root decompression surgery or a narrow lumbar canal and transpedicular fixation. The ACM technique is a midline approach that reduces the number of required holes made in the traditional technique of minimal invasion for the placement of transpedicular screws by half. These holes can be used for the introduction of the endoscopy in the midline to perform root decompression, canal expansion, discectomy, and unilateral and bilateral decompression without using other holes. The traditional open technique for the midline shows greater bleeding, with the MCA technique bleeding reduced by 90%.

In the ACM technique, the recovery time and remission of the symptoms were evaluated by the ODI. The results show that less disability is manifested with rapid remission of symptoms and prescribing patients to perform activities of daily living. Traditional open midline surgery is more traumatic, with greater postoperative pain, longer recovery time, and longer hospital stay.

If reoperation is required, in the ACM, the same approach can be used, both for the removal of screws or, if required, it can be converted into a traditional midline open surgery, joining the holes used with the ACM.

The ODI^[12,13]^ is 1 of the most common scales among research evaluating the effectiveness of treatments and surgeries to manage lower back pain. In addition, the domains evaluated are common activities of daily living, which are characteristics that make it consistent with the goals pursued when medical care is provided. In essence, scales employed to evaluate the evolution of the patient cannot substitute imaging studies (such as radiography and magnetic resonance imaging); they provide anatomical evidence of the mid-term and long-term results of surgery. However, the aim of this study was to evaluate the functionality of patients who underwent endoscopy, which is minimally invasive, and the scales used provided useful results.

The changes in pain reported by the patients immediately after surgery persisted until the first month. This result suggests that the universal approach of full endoscopy and percutaneous transpedicular fixation of the lumbar spine via an ACM is highly effective for patients with lumbar degenerative surgical pathologies. The pain intensity did not change from the first to the third month; however, this finding confirms the rapid improvement after surgery. Importantly, during the first month, the pain reached optimal levels and was maintained over time, which is the desired outcome of any medical management of chronic pain.

With regard to the remaining evaluated domains, clear improvement was observed beginning with the first month after surgery. This finding remained stable at the third month, and clinical improvement was still evident and stable at 12 months. These results indicate that between 6 and 12 months after surgery, the pain level does not significantly change. However, this lack of change was noted because the optimal levels were reached at 6 months and subsequently remained stable. This finding demonstrates the high effectiveness of full endoscopy and percutaneous transpedicular fixation via an ACM among patients with lumbar degenerative surgical pathologies.

The results presented herein emphasize the numerical improvement in the domains evaluated after surgery. Furthermore, the instruments used to evaluate treatment effectiveness demonstrate the specific clinical state for each domain (see Section 3), showing the evident recovery of functionality.

Given these results, the proposed universal approach for endoscopy using transpedicular screws via an ACM (in which unilateral or bilateral decompression and placement of percutaneous transpedicular screws are performed bilaterally through the midline port) reduces the aggravation of the surrounding tissues, largely contributing to the prompt recovery of the patient and the remission of their symptoms.

From a methodological perspective, 1 could criticize the absence of a control group to compare the ACM technique with the traditional approach. Undoubtedly, the ideal is to have 2 groups, but having only 1 does not disqualify the results. The before-after similarity presented in this study is recognized methodologically when referring to causality. In the same sense, 1 might criticize the sample size. It is true that the sample size was not calculated. However, the best evidence that the minimum size complied with the minimum sample size required is that the statistical event was reached between the measurement prior to the surgery and the measurement reported after the surgery, that is, a sample size increasing the probability of not finding significance, a scenario that was not found in this work. It is true that the comparison between the symptoms at 6 months and those at 12 months does not demonstrate significance in the parameters evaluated, which reaffirms the permanence of the effect derived from the ACM.

The 1-year follow-up period of this study is a strong point; although the medical history of the patient must be reviewed over a longer time period, 12 months is substantial enough to offer a broad initial view based on the results shown herein. Above all, it allows us to consider the usefulness and effectiveness of the current surgical technique.

For the patient, being pain-free and recovering the functionality that enables him or her to perform activities of daily living are of utmost importance. If these goals are achieved with full endoscopy and percutaneous transpedicular fixation via an ACM, then this procedure effectively manages patients with lumbar degenerative surgical pathologies.

## 5 Conclusion

The universal approach to full endoscopy and lumbar percutaneous transpedicular fixation via an ACM is highly effective for patients with lumbar surgical degenerative pathologies.

## Author contributions

**Conceptualization:** Víctor Hugo Malo-Camacho, Gerardo Enrique Bañuelos-Díaz, Víctor Hugo Martínez-Velázquez, Luis López-Ortega, Óscar Malo-Macias, Enrique Villarreal-Ríos, Alejandro Sosa-Gallegos, Mario Iván Mejía-Valencia.

**Data curation:** Víctor Hugo Malo-Camacho, Gerardo Enrique Bañuelos-Díaz, Víctor Hugo Martínez-Velázquez, Luis López-Ortega, Óscar Malo-Macias, Enrique Villarreal-Ríos, Alejandro Sosa-Gallegos, Mauricio Alva-Nájera, Mario Iván Mejía-Valencia.

**Formal analysis:** Víctor Hugo Malo-Camacho, Gerardo Enrique Bañuelos-Díaz, Víctor Hugo Martínez-Velázquez, Luis López-Ortega, Óscar Malo-Macias, Enrique Villarreal-Ríos, Alejandro Sosa-Gallegos, Mauricio Alva-Nájera, Mario Iván Mejía-Valencia.

**Funding acquisition:** Víctor Hugo Malo-Camacho.

**Investigation:** Víctor Hugo Malo-Camacho, Gerardo Enrique Bañuelos-Díaz, Enrique Villarreal-Ríos, Mauricio Alva-Nájera.

**Methodology:** Víctor Hugo Malo-Camacho, Gerardo Enrique Bañuelos-Díaz, Víctor Hugo Martínez-Velázquez, Luis López-Ortega, Óscar Malo-Macias, Enrique Villarreal-Ríos, Alejandro Sosa-Gallegos, Mauricio Alva-Nájera, Mario Iván Mejía-Valencia.

**Project administration:** Víctor Hugo Malo-Camacho, Alejandro Sosa-Gallegos.

**Resources:** Víctor Hugo Malo-Camacho.

**Supervision:** Víctor Hugo Malo-Camacho, Víctor Hugo Martínez-Velázquez, Luis López-Ortega, Óscar Malo-Macias, Mario Iván Mejía-Valencia.

**Validation:** Víctor Hugo Malo-Camacho, Gerardo Enrique Bañuelos-Díaz, Víctor Hugo Martínez-Velázquez, Luis López-Ortega, Óscar Malo-Macias, Enrique Villarreal-Ríos, Alejandro Sosa-Gallegos, Mauricio Alva-Nájera, Mario Iván Mejía-Valencia.

**Visualization:** Víctor Hugo Malo-Camacho.

**Writing – original draft:** Víctor Hugo Malo-Camacho, Gerardo Enrique Bañuelos-Díaz, Víctor Hugo Martínez-Velázquez, Luis López-Ortega, Óscar Malo-Macias, Enrique Villarreal-Ríos, Alejandro Sosa-Gallegos, Mauricio Alva-Nájera, Mario Iván Mejía-Valencia.

**Writing – review & editing:** Víctor Hugo Malo-Camacho, Gerardo Enrique Bañuelos-Díaz, Víctor Hugo Martínez-Velázquez, Luis López-Ortega, Óscar Malo-Macias, Enrique Villarreal-Ríos, Alejandro Sosa-Gallegos, Mauricio Alva-Nájera, Mario Iván Mejía-Valencia.
